# Supplementary material for: A Rearrangement of the Mitochondrial Genes of Centipedes (Arthropoda, Myriapoda) with a Phylogenetic Analysis
Source: Genes (Basel). 2022 Oct 3;13(10):1787. doi: 10.3390/genes13101787 (PMC9601646; doi:10.3390/genes13101787)
Supplement: Supplementary file 1 [file genes-13-01787-s001.zip › genes-1933801-supplementary.pdf]

genes

Supplementary Tables and Figures

A Rearrangement of the Mitochondrial Genes of Centipedes (Arthropoda, Myriapoda) with a Phylogenetic Analysis

JiaJia Wang, Yu Bai and Yan Dong *

College of Biology and Food Engineering, Chuzhou University, Chuzhou, China
* Correspondence: dongyan_bio@126.com

Table S1. GenBank accession numbers for taxa used in this study.

Taxon	Species	Accession No.	References
Chilopoda	*Lithobius forficatus*	NC_002629	[1]
	Bothropolys sp.	NC_009458	[2]
	Cermatobius longicornis	NC_021403	[3]
	Scutigera coleoptrata	NC_005870	[4]
	Scolopendra dehaani	KY947341	[5]
	Scolopendra mutilans	MN317390	[6]
	Scolopendra subspinipes	MN642577	this study
	Scolopocryptops sp.	KC200076	[7]
	Strigamia maritima	NC_026557	[8]
	Mecistocephalus marmoratus	KX774322	this study
	Scolopendra morsitans	MW810062	[27]
	Scolopocryptops sp.	KC200076	[28]
Diplopoda	*Narceus annularus*	NC_003343	[9]
	Thyropygus sp.	NC_003344	[9]
	Antrokoreana gracilipes	NC_010221	[10]
	Abacion magnum	NC_021932	[11]
	Brachycybe lecontii	NC_021934	[11]
	Appalachioria falcifera	NC_021933	[11]
	Asiomorpha coarctata	KU721885	[12]
	Xystodesmus sp.	KU721886	[12]
Symphyla	*Scutigerella causeyae*	NC_008453	[13]
	Symphylella sp.	NC_011572	[14]
Pauropoda	*Pauropus longiramus*	NC_016676	[15]
Chelicerata	*Achella bituberculata*	NC_009724	[16]
	Calisoga longitemsis	NC_010780	[17]
	Limulus polyphemus	NC_003057	[18]
	Tachyleus tridentatus	NC_012574	[19]
	Phalangium opilio	NC_010766	[17]
Crustacea	*Penaeus monodon*	NC_002184	[20]
	Hutchinsoniella macracantha	NC_005937	[21]
Hexapoda	*Petrobius brevistylis*	NC_007688	[22]
	Thermobia domestica	NC_006080	[23]
	Drosophila yakuba	NC_001322	[24]
	Japyx solifugus	NC_007214	[25]
Onychophora	*Epiperipatus biolleyi*	NC_009082	[13]
Priapulida	*Priapulus caudatus*	NC_008557	[26]

References

1. Lavrov, D.V.; Brown, W.M.; Boore, J.L. A novel type of RNA editing occurs in the mitochondrial tRNAs of the centipede Lithobius forficatus. *Proc. Natl. Acad. Sci. USA* **2000**, *97*, 13738–13742.
2. Park, S.J.; Choi, E.H.; Hwang, J.S.; Hwang, U.W. The complete mitochondrial genome of a centipede *Bothropolys* sp. (Chilopoda, Lithobiomorpha, Lithobiidae). *Mitochondrial DNA Part A* **2016**, *27*, 2268–2269.
3. Gai, Y.; Ma, H.; Sun, X.; Ma, J.; Li, C.; Yang, Q. The complete mitochondrial genome of *Cermatobius longicornis* (Chilopoda: Lithobiomorpha: Henicopidae). *Mitochondrial DNA* **2013**, *24*, 331–332.
4. Negrisolo, E.; Minelli, A.; Valle, G. The mitochondrial genome of the house centipede *Scutigera* and the monophyly versus paraphyly of myriapods. *Mol. Biol. Evol.* **2004**, *21*, 770–780.
5. Sun, L.; Qi, Y.; Tian, X. Analysis of mitochondrial genome of *Scolopendra subspinipes dehaani*. *Tianjin J. Tradit. Chin. Med.* **2018**, *35*, 225–229.
6. Hu, C.; Wang, S.; Huang, B.; Liu, H.; Xu, L.; Hu, Z.; Liu, Y. The complete mitochondrial genome sequence of *Scolopendra mutilans* L. Koch, 1878 (Scolopendromorpha, Scolopendridae), with a comparative analysis of other centipede genomes. *ZooKeys* **2020**, *925*, 73–88.
7. Gai, Y.; Ma, H.; Ma, J.; Li, C.; Yang, Q. The complete mitochondrial genome of *Scolopocryptops* sp. (Chilopoda: Scolopendromorpha: Scolopocryptopidae). *Mitochondrial DNA* **2014**, *25*, 192–193.
8. Robertson, H.E.; Lapraz, F.; Rhodes, A.C.; Telford, M.J. The complete mitochondrial genome of the geophilomorph centipede *Strigamia maritima*. *PLoS ONE* **2015**, *10*, e0121369.
9. Lavrov, D.V.; Boore, J.L.; Brown, W.M. Complete mtDNA sequences of two *millipedes* suggest a new model for mitochondrial gene rearrangements: duplication and nonrandom loss. *Mol. Biol. Evol.* **2002**, *19*, 163–169.
10. Woo, H.J.; Lee, Y.S.; Park, S.J.; Lim, J.T.; Jang, K.H.; Choi, E.H.; Choi, Y.G.; Hwang, U.W. Complete mitochondrial genome of a troglobite millipede *Antrokoreana gracilipes* (Diplopoda, Juliformia, Julida), and juliformian phylogeny. *Mol. Cells* **2007**, *23*, 182–191.
11. Brewer, M.S.; Swafford, L.; Spruill, C.L.; Bond, J.E. Arthropod phylogenetics in light of three novel *millipede* (Myriapoda: Diplopoda) mitochondrial genomes with comments on the appropriateness of mitochondrial genome sequence data for inferring deep level relationships. *PLoS ONE* **2013**, *8*, e68005.
12. Dong, Y.; Zhu, L.; Bai, Y.; Ou, Y.; Wang, C. Complete mitochondrial genomes of two flat-backed *millipedes* by next-generation sequencing (Diplopoda, Polydesmida). *ZooKeys* **2016**, *637*, 1–20.
13. Podsiadlowski, L.; Kohlhagen, H.; Koch, M. The complete mitochondrial genome of *Scutigerella causeyae* (Myriapoda: Symphyla) and the phylogenetic position of Symphyla. *Mol. Phylogenet. Evol.* **2007**, *45*, 251–260.
14. Gai, Y.; Song, D.; Sun, H.; Yang, Q.; Zhou, K. The complete mitochondrial genome of *Symphylella* sp.(Myriapoda: Symphyla): Extensive gene order rearrangement and evidence in favor of Progoneata. *Mol. Phylogenet. Evol.* **2008**, *49*, 574–585.
15. Dong, Y.; Sun, H.; Guo, H.; Pan, D.; Qian, C.; Hao, S.; Zhou, K. The complete mitochondrial genome of *Pauropus longiramus* (Myriapoda: Pauropoda): Implications on early diversification of the myriapods revealed from comparative analysis. *Gene* **2012,** *505*, 57–65.
16. Park, S.J.; Lee, Y.S.; Hwang, U.W. The complete mitochondrial genome of the sea spider *Achelia bituberculata* (Pycnogonida, Ammotheidae): arthropod ground pattern of gene arrangement. *BMC Genom.* **2007**, *8*, 343.
17. Masta, S.E.; Boore, J.L. Parallel evolution of truncated transfer RNA genes in arachnid mitochondrial genomes. *Mol. Biol. Evol.* **2008**, *25*, 949–959.
18. Lavrov, D. V.; Boore, J. L.; Brown, W. M. The complete mitochondrial DNA sequence of the horseshoe crab Limulus polyphemus. *Mol. Biol. Evol.* **2000**, *17*, 813–824.
19. Shingate, P.; Ravi, V.; Prasad, A.; Tay, B.H.; Venkatesh, B. Chromosome-level genome assembly of the coastal horseshoe crab (Tachypleus gigas). *Mol. Ecol. Resour.* **2020**, *20*, 1748–1760.
20. Wilson, K.; Cahill, V.; Ballment, E.; Benzie, J. The complete sequence of the mitochondrial genome of the crustacean *Penaeus monodon*: are malacostracan crustaceans more closely related to insects than to branchiopods? *Mol. Biol. Evol.* **2000**, *17*, 863–874.
21. Lavrov, D.V.; Brown, W.M.; Boore, J.L. Phylogenetic position of the Pentastomida and (pan) crustacean relationships. *Proc. R. Soc. London. Ser. B Biol. Sci.* **2004**, *271*, 537–544.
22. Podsiadlowski, L. The mitochondrial genome of the bristletail *Petrobius brevistylis* (Archaeognatha: Machilidae). *Insect Mol. Biol.* **2006**, *15*, 253–258.
23. Cook, C.E.; Yue, Q.; Akam, M. Mitochondrial genomes suggest that hexapods and crustaceans are mutually paraphyletic. *Proc. R. Soc. B Biol. Sci.* **2005**, *272*, 1295–1304.
24. Clary, D.O.; Wolstenholme, D.R. The ribosomal RNA genes of *Drosophila* mitochondrial DNA. *Nucleic Acids Res.* **1985**, *13*, 4029–4045.
25. Carapelli, A.; Nardi, F.; Dallai, R.; Boore, J.; Lio, P.; Frati, F. Relationships between hexapods and crustaceans based on four mitochondrial genes. *Crustacean Issues* **2005,** *16*, 295.
26. Webster, B.L.; Copley, R.R.; Jenner, R.A.; Mackenzie-Dodds, J.A.; Bourlat, S.J.; Rota-Stabelli, O.; Littlewood, D.; Telford, M.J. Mitogenomics and phylogenomics reveal priapulid worms as extant models of the ancestral Ecdysozoan. *Evol. Dev.* **2006**, *8*, 502–510.
27. Ding, J.; Lan, H.; Xu, W.; Chen, Y.N.; Wu, H.; Jiang, H.M.; Wang, J.C.; Wu, Y.B.; Liu, H. Two complete mitochondrial genomes in *Scolopendra* and a comparative analysis of tRNA rearrangements in centipedes. *Mol. Biol. Rep.* **2022**, *49*, 6173–6180.

28. Gai, Y.; Ma, H.; Ma, J.; Li, C.; Yang, Q. The complete mitochondrial genome of *Scolopocryptops* sp. (Chilopoda: Scolopendromorpha: Scolopocryptopidae). *Mitochondrial DNA* **2014**, *25*, 192–193.

Table S2. Optimal partition strategy and evolutionary models used in phylogenetic analyses.

Dataset	Subset	Best Model	Sites	Partiotion Names
AA	1	MTART+I+G+F	646	atp8, atp6, nad3, nad2, nad6
	2	MTART+I+G	493	cox1
	3	MTART+I+G	452	cox3, cox2
	4	MTART+I+G	322	cob
	5	MTART+I+G+F	1,024	nad5, nad4, nad1, nad4L
PCG12RNA	1	GTR+I+G	721	cox2_pos1, cox1_pos1
	2	GTR+I+G	504	cox1_pos2
	3	TVM+I+G	293	nad1_pos1
	4	GTR+I+G	377	nad4L_pos2, nad1_pos2
	5	GTR+I+G	1,065	cox2_pos2, cob_pos2, cox3_pos2, atp6_pos2
	6	GTR+I+G	559	atp8_pos1, nad6_pos1, nad3_pos1, nad2_pos1
	7	TVM+I+G	559	atp8_pos2, nad6_pos2, nad2_pos2, nad3_pos2
	8	GTR+I+G	848	atp6_pos1, cox3_pos1, cob_pos1
	9	GTR+I+G	1027	nad4L_pos1, nad5_pos1, nad4_pos1
	10	GTR+I+G	943	nad4_pos2, nad5_pos2
	11	GTR+I+G	1,647	rrnL, rrnS
PCGRNA	1	GTR+I+G	721	cox2_pos1, cox1_pos1
	2	GTR+I+G	504	cox1_pos2
	3	GTR +G	1,347	cox2_pos3, cox1_pos3, cox3_pos3, cob_pos3
	4	TVM+I+G	908	rrnS, nad1_pos1
	5	GTR+I+G	377	nad1_pos2, nad4L_pos2
	6	TVM+G	1,320	nad4_pos3, nad4L_pos3, nad5_pos3, nad1_pos3
	7	GTR+I+G	1,065	cox2_pos2, cob_pos2, atp6_pos2, cox3_pos2
	8	GTR+I+G	781	nad3_pos1, nad2_pos1, atp6_pos1, nad6_pos1, atp8_pos1
	9	TVM+I+G	559	atp8_pos2, nad6_pos2, nad3_pos2, nad2_pos2
	10	GTR+G	781	nad2_pos3, nad3_pos3, atp6_pos3, nad6_pos3, atp8_pos3
	11	GTR+I+G	626	cob_pos1, cox3_pos1
	12	GTR+I+G	1,027	nad4L_pos1, nad5_pos1, nad4_pos1
	13	GTR+I+G	943	nad5_pos2, nad4_pos2
	14	GTR+I+G	1,032	rrnL

Table S3. Mitochondrial genomes Organization of *Scolopendra subspinipes.*

Gene	Strand	Location	Length	Start /Stop Codon	Intergenic Nucleotides
trnI	F	1-61	61		-2
trnQ	R	60-129	70		-3
trnM	F	127-194	68		-1
nad2	F	194-1,163	970	ATT/T--	0
trnW	F	1,164-1242	79		-27
trnC	R	1,216-1,277	62		-4
trnY	R	1,274-1,334	61		-6
cox1	F	1,299-2,864	1,566	TTA/TAA	3
cox2	F	2,868-3,539	672	ATG/TAA	-7
trnK	F	3,538-3,604	67		0
trnD	F	3,604-3,664	61		-1
atp8	F	3,664-3,819	156	ATT/TAA	-7
atp6	F	3,816-4,479	667	ATA/T--	0
cox3	F	4,480-5262	783	ATG/TAA	4
trnG	F	5,267-5,330	64		-1
nad3	F	5,330-5,678	349	ATA/T--	0
trnA	F	5,679-5,740	62		6
trnR	F	5,747-5,806	60		1
trnN	F	5,808-5,863	56		-3
trnS1	F	5,861-5,912	52		-1
trnE	F	5,912-5,975	64		-5
trnF	R	5,971-6,031	61		2
nad5	R	6,034-7,732	1,699	TTA/TAA	1
trnH	R	7,734-7,794	61		1
nad4	R	7,794-9,125	1,332	ATG/T	-7
nad4L	R	9,119-9,403	285	ATA/TAA	7
trnT	F	9,411-9,472	62		-2
trnP	R	9,471-9,528	58		2
nad6	F	9,525-9,998	474	ATT/TAA	47
cob	F	10,044-11,155	1,112	ATG/TAA	0
trnS2	F	11,156-11,221	66		1
nad1	R	11,223-12,140	918	ATT/TAG	1
trnL2	R	12,142-12,203	62		-9
trnL1	R	12,195-12,254	60		0
rrnL	R	12,255-13,398	1,144		0
trnV	F	13,399-13,458	60		0
rrnS	R	13,459-13,918	460		0
CR	F	13,919-14,637	719		0

Table S4. Mitochondrial genomes Organization of *Mecistocephalus marmoratus.*

Gene	Strand	Location	Length	Start /Stop Codon	Intergenic Nucleotides
cox1	F	1-1,536	1,536	ATA/TAA	0
cox2	F	1,536-2,216	681	ATG/TAA	-1
trnK	F	2,215-2279	65		-2
trnD	F	2,281-2,342	62		1
atp8	F	2,343-2,498	156	ATT/TAA	0
atp6	F	2,492-3,160	669	ATG/TAA	-7
cox3	F	3,160-3,945	786	ATG/TAA	-1
trnG	F	3,945-4,005	61		-1
trnA	F	4,006-4,065	60		0
trnR	F	4,067-4,124	58		1
trnS1	F	4,129-4,187	59		4
trnE	F	4,195-4,251	57		7
trnT	F	4,255-4,306	52		3
nad6	F	4,311-4,814	504	ATA/TAA	4
cob	F	4,818-5,954	1,137	ATG/TAA	3
trnS2	R	5,954-6,024	71		-1
trnQ	F	6,023-6,091	69		-2
trnM	F	6,083-6,147	65		-9
nad2	F	6,148-7,137	990	ATC/TAA	0
trnW	F	7,136-7,201	66		-2
nad3	F	7,208-7,561	354	ATG/TAA	6
trnN	F	7,561-7,633	73		-1
trnF	R	7,627-7,689	63		-7
nad5	R	7,688-9,382	1,695	ATT/TAA	-2
trnH	R	9,390-9,451	62		7
nad4	R	9,452-10,775	1,324	ATG/T--	0
nad4L	R	10,769-11,047	279	ATT/TAG	-7
trnP	R	11,051-11,111	61		3
nad1	R	11,112-12,036	925	ATA/T--	0
trnL2	R	12,038-12,099	62		1
rrnL	R	12,100-13,414	1,315		0
trnV	R	13,415-13,481	67		0
rrnS	R	13,482-14,246	765		0
trnL1	R	14,247-14,317	71		0
CR	F	14,318-15,080	766		0
trnC	F	15,084-15,137	54		0
trnY	R	15,145-15,209	65		7
trnI	F	15,211-15,277	67		1

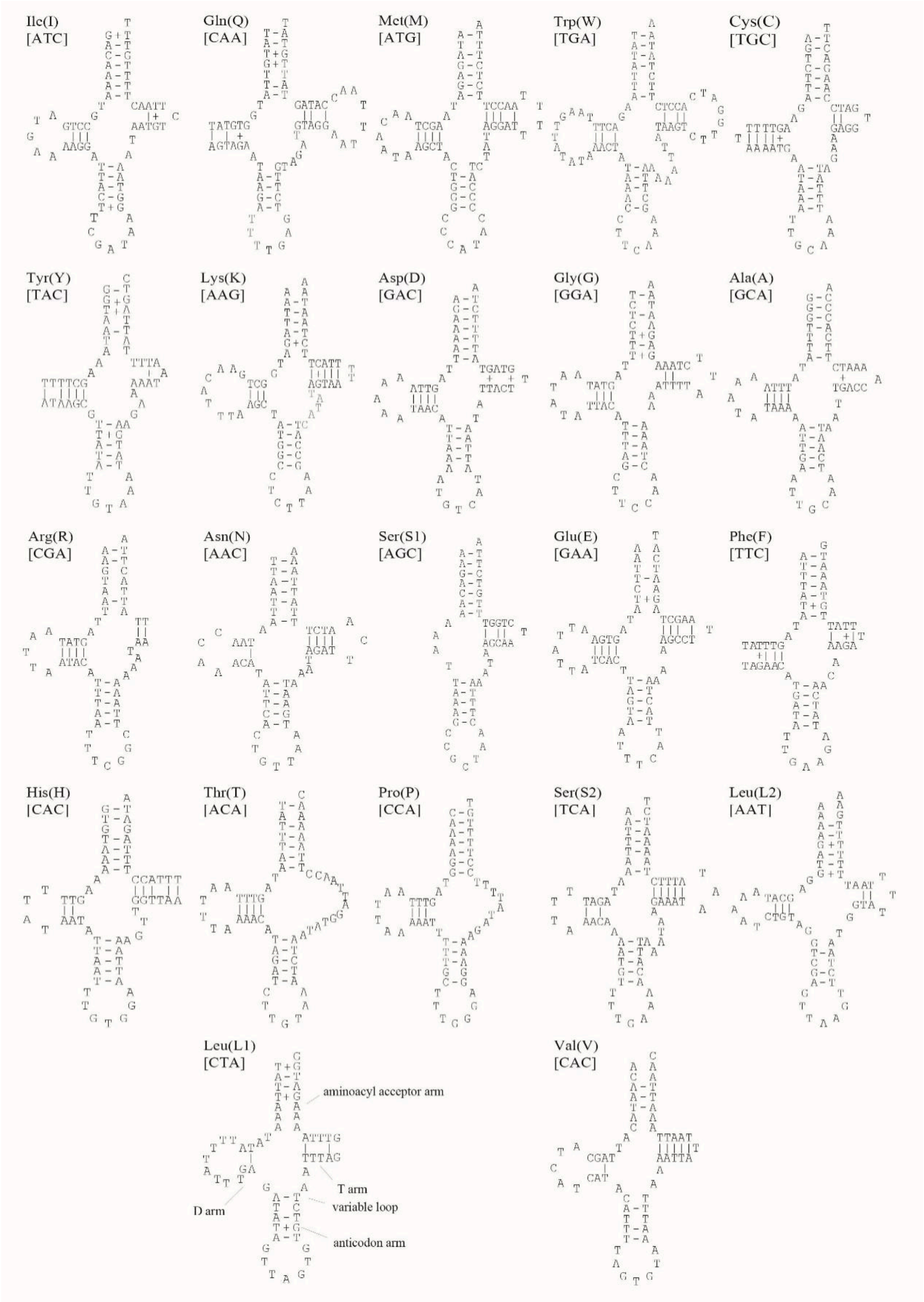

Figure S1. Predicted secondary structure of the 22 tRNAs in the *Scolopendra subspinipes* mitogenome Dashes (−) indicate Watson–Crick base pairing.

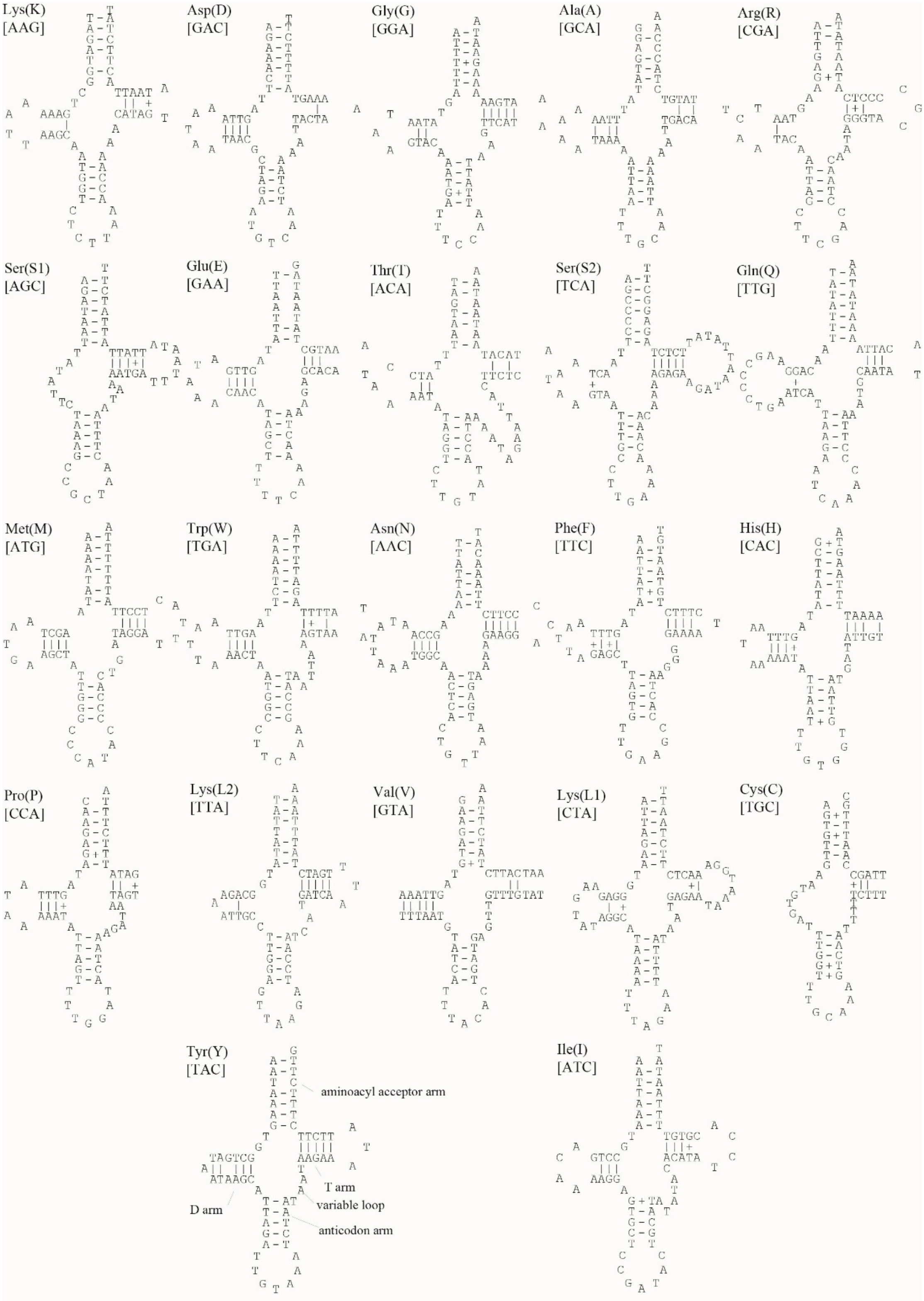

Figure S2. Predicted secondary structure of the 22 tRNAs in the *Mecistocephalus marmoratus* mitogenome Dashes (−) indicate Watson–Crick base pairing.
